# The Annual Commencement of the Buffalo Medical College

**Published:** 1887-04

**Authors:** 


### Editorial.

### *THE ANNUAL COMMENCEMENT OF THE BUFFALO MEDICAL COLLEGE.*

The commencement exercises of the college took place this year on March 1st. The first part of the day was occupied by the meeting of the alumni as is usual. About forty of the alumni responded to the call of their executive committee; eight from neighboring towns, the rest from this city. They assembled at the college at 11 A. M. The president, Dr. Conrad Diehl, called the meeting to order, and in a few well-chosen words introduced the order of business. Prof. Pohlman of the faculty delivered the address of welcome. It was not up to the usual standard of the doctor's contributions.

In the afternoon, papers were read by Dr. Roswell Park, Dr. M. D. Mann, Dr. John H. Pryor and Dr. Wende. There was a somewhat increased attendance, and the papers were well received. The following officers were elected:

*President*, Henry Lapp, Clarence; *Vice-President*, D. W. Harrington, Buffalo; *Second Vice-President*, C. S. Pugsley, Oakfield; *Third Vice-President*, C. A. Ring, Buffalo; *Fourth Vice-President*, A. G. Ellenwood, Attica; *Fifth Vice-President*, Miss Bianca Potter, Buffalo; *Permanent Secretary*, J. J. Walsh, Buffalo; *Recording Secretary*, W. H. Thornton, Buffalo; *Treasurer*, E. C. W. O'Brien, Buffalo; *Trustees*, Henry Lapp, Clarence; P. W. Van Peyma, Buffalo; E. C. W. O'Brien, Buffalo; C. Diehl, Buffalo; H. Hoyt, East Aurora; *Executive Committee*, F. P. Vandenbergh, J. H. Pryor, H. H. Bingham, Buffalo; ex-officio, Prof. Chas. Cary, C. H. Lapp; *Committee on Nominations*, Dr. E. C. W. O'Brien, James S. Porter, B. G. Long, F. E. L. Brecht, F. O. Vaughn.

The council held a meeting in the afternoon. The committee appointed to establish a law school reported that while this was a favorable point for such a school, they had been unable to secure the men necessary to conduct it. The following degrees were conferred. Degree of M. D. on—

William W. Ruby, Rochester, N. Y.; Elizabeth Johnson, Montreal, Canada; Brayton Nelson Strong, Colden, N. Y.; William C. L. Meisburger, Buffalo, N. Y.; James C. Earle, Belfast, N. Y.; Earl A. Schofield, Gerry, N. Y.; Harvey E. Brown, Fayette, N. Y.; Charles Davis Johnson, Hartstown, Pa.; Edward Meany, Jacksonville, N. Y.; William Harry Bergtold, Buffalo, N. Y.; Frederick Hill Stanbro, Springville, N. Y.; William Henry Olmsted, Elmira, N. Y.; Bernard Cohen, Buffalo, N. Y.; George Mark Harrison, Dunkirk, N. Y.; William Holden Chace, A. B., Mayville, N. Y.; Clark Francis Bruso, Buffalo, N. Y.; Thomas Ed. Soules, Ph. G., Cherry Creek, N. Y.; Fridoin Thoma, Buffalo, N. Y.; Arthur Wm. Hubbard, Punxsutawney, Pa.; Stella Cox Venable, Geneseo, N. Y.; Alexander McNamara, Corning, N. Y.; La Rue R. Colgrove, Ph. G., Elmira, N. Y.; Jacob W. E. K. Davis, Easton, Pa.; Franklin Ayer Trippett, Williamsville, N. Y.; David H. Webster, New Buro, Canada; Clarence Victor Gray, Hulberton, N. Y.; Clifton George Smith, Carlton, N. Y.; Frank Wesley Huff, Penn Yan, N. Y.; Burg Chadwick, Smethport, Pa.; Charles W. Davis, Randolph, N. Y.; Andrew J. Martin, Clarence Centre, N. Y.; William H. Mansperger, Buffalo, N. Y.; George W. Roos, Buffalo, N. Y.; Marshall C. Butler, Little Valley, N. Y.; Mark Alward, Petitcodiac, Canada; Edward H. Wells, Geneva, N. Y.; Jasper D. Wooster, Buffalo, N. Y.; George Stephen Skiff, Pike, N. Y.; Joseph W. Magill, Pittsford, N. Y.; Alfred Day, Rochester, N. Y.; Jeremiah J. Sullivan, Batavia, N. Y.; Adolph A. Auger, Roxton Pond, Canada; George H. Sisson, Buffalo, N. Y.; Philo L. Alden, Pultney, N. Y.; Guy Bradford Crandall, Randolph, N. Y.; Peter Guinan, Mendon, N. Y.; Grosvenor Reuben Trowbridge, A. B., Buffalo, N. Y.; William Russel Palmer, Port Allegheny, Pa.; Eugene A. Smith, Buffalo, N. Y.; William W. Skinner, Prattsburgh, N. Y.

The degree of Bachelor of Science on Dr. F. P. Vandenbergh, assistant professor of pharmaceutical chemistry; degree of Ph. G. on Willis G. Gregory. Curators of the college, Dr. B. H. Putnam, of North East; W. E. Landerdale, of Geneseo, and Dr. S. C. Pugsley, of Oakfield; and C. H. Haskins, of Rochester, and J. P. Diehl, of Buffalo, curators of college of pharmacy in place of G. H. Haas, resigned, and J. Rieffenstahl, deceased.

### THE COMMENCEMENT.

The graduating exercises took place in the evening at Liedertafel Hall, with a large attendance. The degrees were conferred as above by the chancellor. Honorable mention was made of the graduates, and their standing in the class announced as follows:

Eugene A. Smith, George M. Harrison, William H. Chace, William W. Skinner, La Rue C. Colegrove, William W. Ruby, Edward Meany, James C. Earle, Clark F. Bruso, Stella Cox Venable.

The following theses were considered worthy of honorable mention: "The Third State of Anæsthesia," by Eugene A. Smith. "A Comparative Test of Pepsin Preparations," by Miss Elizabeth Johnson. "Quantitative Analysis of Urine in the Diagnosis of Malignant Tumors," by Jasper D. Wooster.

The address to the graduating class was delivered by Prof. J. M. Cassety, principal of the Normal School, and was eloquent and interesting. The responsibilities of the medical profession were shown at length: first, in its relation to science; second to law; third, to humanity. The sacred duties of the physician in the family and in the presence of death were fittingly and forcibly described.

The address to the alumni was delivered by Dr. P. M. Wise, superintendent of the Willard Insane Asylum .

